# Lymphoma masquerading as Paget’s disease of bone: a rare diagnostic challenge

**DOI:** 10.1007/s00256-025-05003-3

**Published:** 2025-09-03

**Authors:** Irina D. Sokolik, Timothy A. Damron

**Affiliations:** 1 Upstate Department of Orthopedic Surgery, Upstate Medical University, 750 E Adams St, Syracuse, NY 13210 USA; 2 Upstate Department of Orthopedic Surgery, Upstate Medical University, Upstate Bone and Joint Center, 6620 Fly Road, East Syracuse, NY 13057 USA

**Keywords:** Lymphoma, Paget’s disease of bone, Femur, Radiographic imaging, MRI

## Abstract

Paget’s disease of bone (PDB) is a skeletal remodeling disorder diagnosed primarily via radiographs. In long bones, the early lytic stage of the disease is characterized by flame-shaped or blade of grass radiolucent bone resorption beginning in subchondral bone with variable length of extension into the metadiaphysis, and the later stages show bone expansion, cortical thickening, and coarsening of the trabeculae [1, 2]. Despite the usually diagnostic features, other considerations with overlapping appearance include aggressive benign and malignant bone tumors [3]. Malignancy such as lymphoma can present shared clinical features to PDB. When there is doubt as to the diagnosis, biopsy should be performed. A 60-year-old male presented with chronic left lower extremity pain. Radiographs showed a flame-shaped lytic lesion in the left femur with corresponding uptake on the bone scintigraphy. Radiographic features were suggestive of PDB, but due to some atypical findings, a biopsy was performed and showed small lymphocytic lymphoma (SLL) in the setting of chronic lymphocytic leukemia (CLL). This diagnosis led to the patient being promptly treated with targeted therapy and radiation. Pathologic verification is critical in an aberrant presentation of PDB to mitigate misdiagnosis and establish an appropriate therapeutic course.

## Introduction

Paget’s disease of bone (PDB) is a metabolic skeletal disease characterized by irregular bone remodeling, where lamellar bone is excessively resorbed and replaced with sclerotic woven bone that is substantially weaker. Patients often present with localized pain, pathological fractures, and hearing loss if the disease involves the skull. Imaging often shows a combination of lytic and sclerotic lesions, but the characteristic radiographic features are bone expansion, cortical thickening, and coarsening of the trabeculae [1].

The clinical and radiographic appearance of lymphoma overlaps with those of PDB, complicating diagnostic decisions and treatment management. Lymphoma affecting bone can also display pain, lytic lesions, and pathologic fractures [4]. This case report depicts a unique presentation of small lymphocytic lymphoma (SLL) simulating PDB, accentuating diagnostic challenges, and highlighting the necessity of considering histopathologic confirmation to avoid misdiagnosis and delaying appropriate treatment.

## Case report

A 60-year-old male presented with a 6-month history of chronic left lower extremity pain not preceded by injury. Pain was exacerbated by weight-bearing, but there was no rest pain or night-awakening pain. There was no pertinent past medical history or laboratory values; serum alkaline phosphatase was within normal range.

**Fig. 1:**
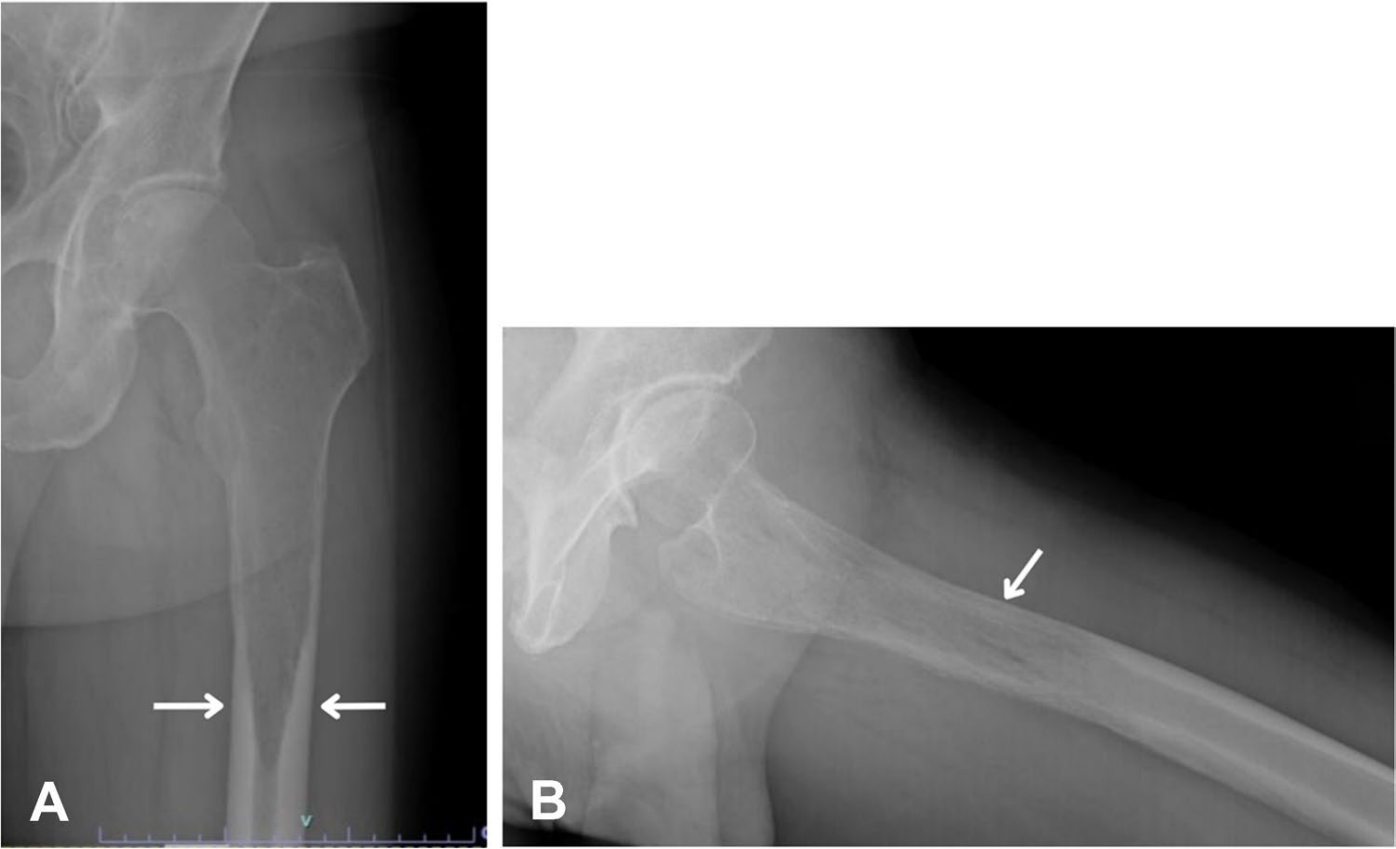
**A** Anteroposterior radiograph demonstrates a flame shape (blade of grass—arrows) lytic lesion in the proximal diaphysis, initially attributed to the lytic phase of Paget’s disease of bone (PDB). **B** Lateral radiograph of the proximal femur shows osteolysis, significant endosteal scalloping and coarsened trabeculae also suggesting PDB (arrow)

**Fig. 2:**
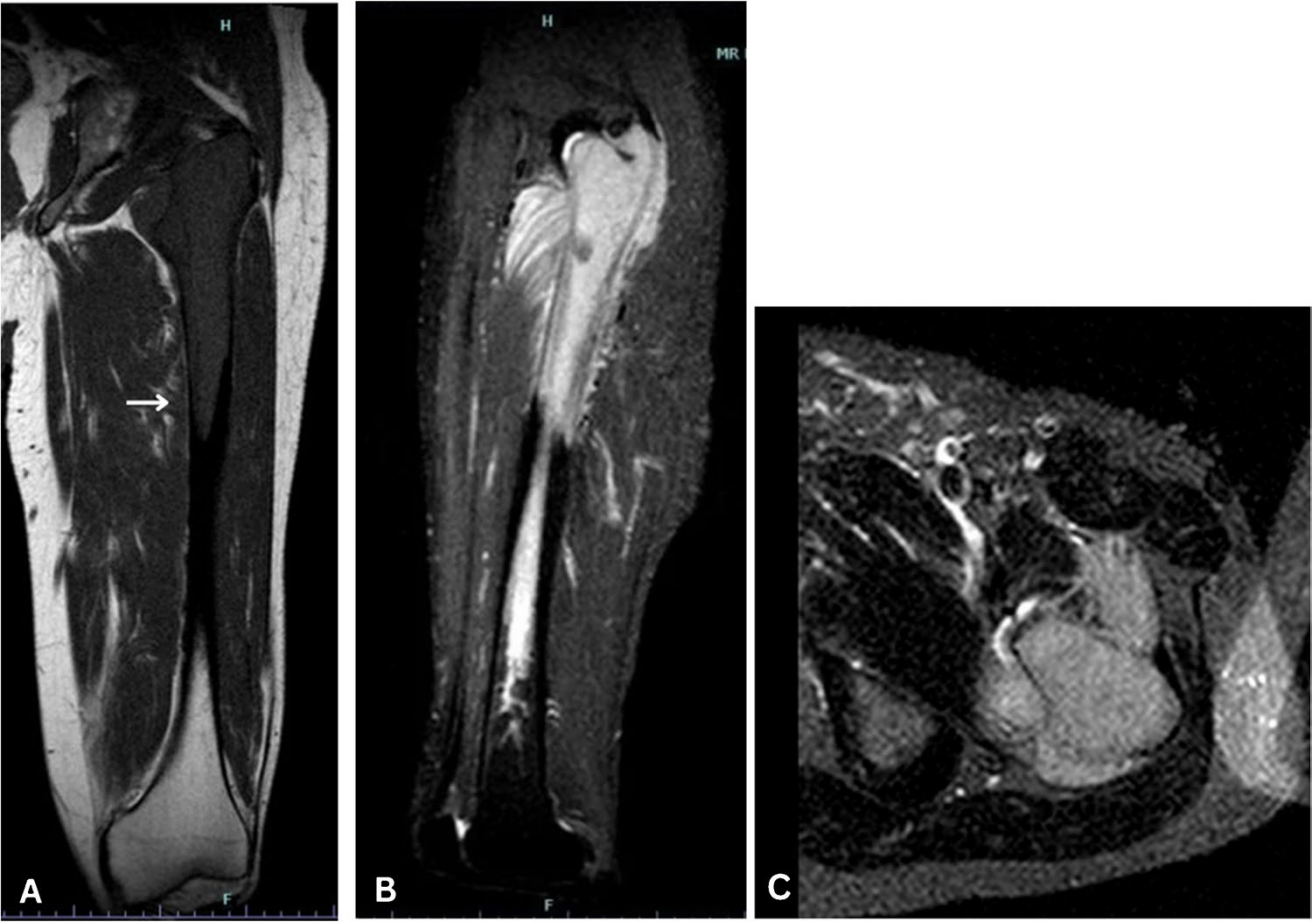
**A** Coronal T1W TSE—no contrast (TR353.77, TE15); diffuse marrow replacement, cortical erosion and abnormal signal in the adjacent proximal thigh soft tissues suggesting extension of the primary process and increasing suspicion for an aggressive neoplasm. **B** Sagittal STIR TSE—no contrast (TR3000, TE20). **C** Axial STIR MT—no contrast (TR3000, TE80) soft tissue extension (arrow) demonstrated

**Fig. 3:**
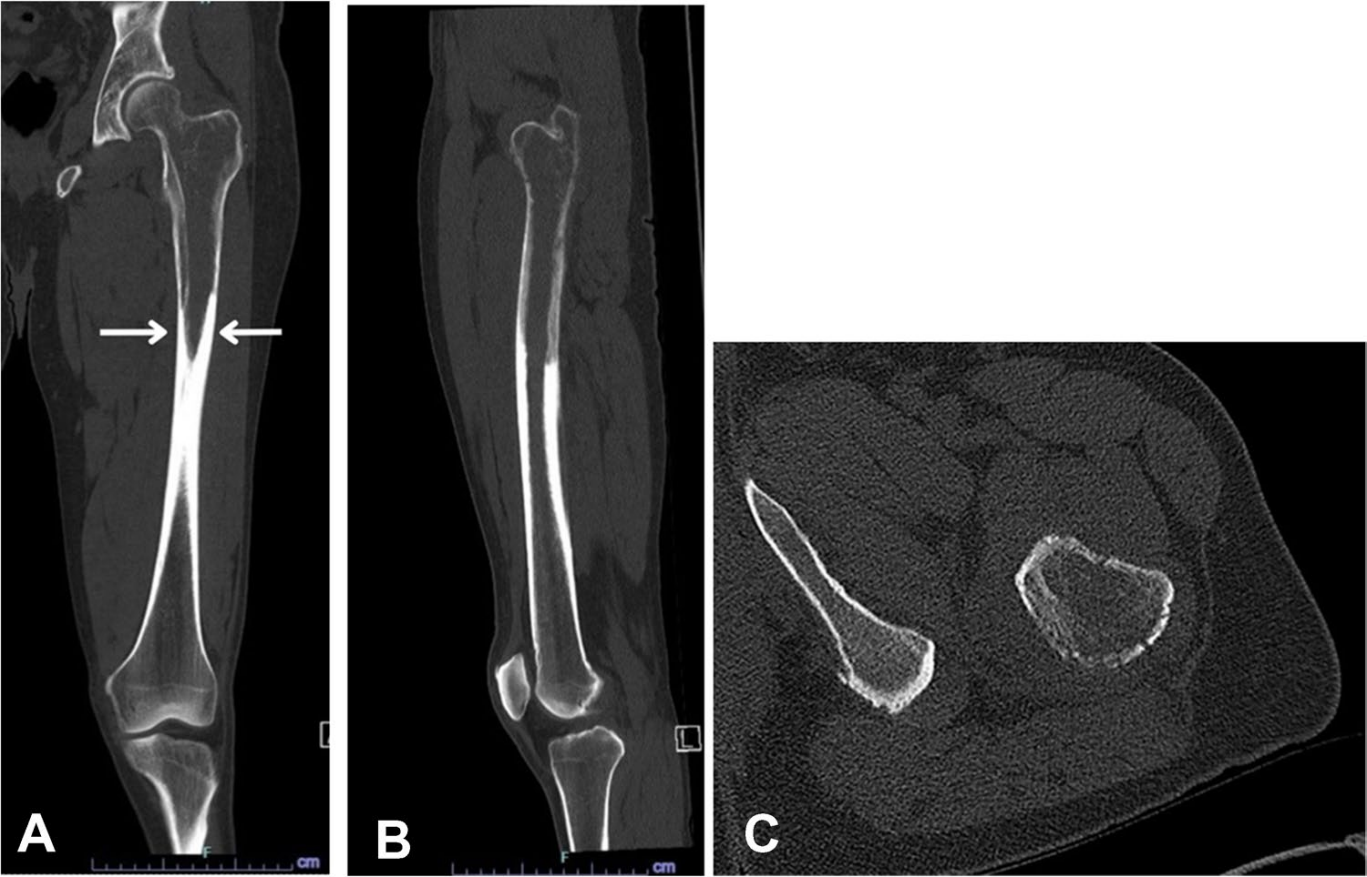
Coronal (**A**) and sagittal (**B**) CT images show cortical thickening with intracortical lucency and flame shape along the proximal diaphysis (arrows) and axial CT (**C**) also reveals the osteolytic lesion with permeative loss of definition of the surrounding cortex

**Fig. 4:**
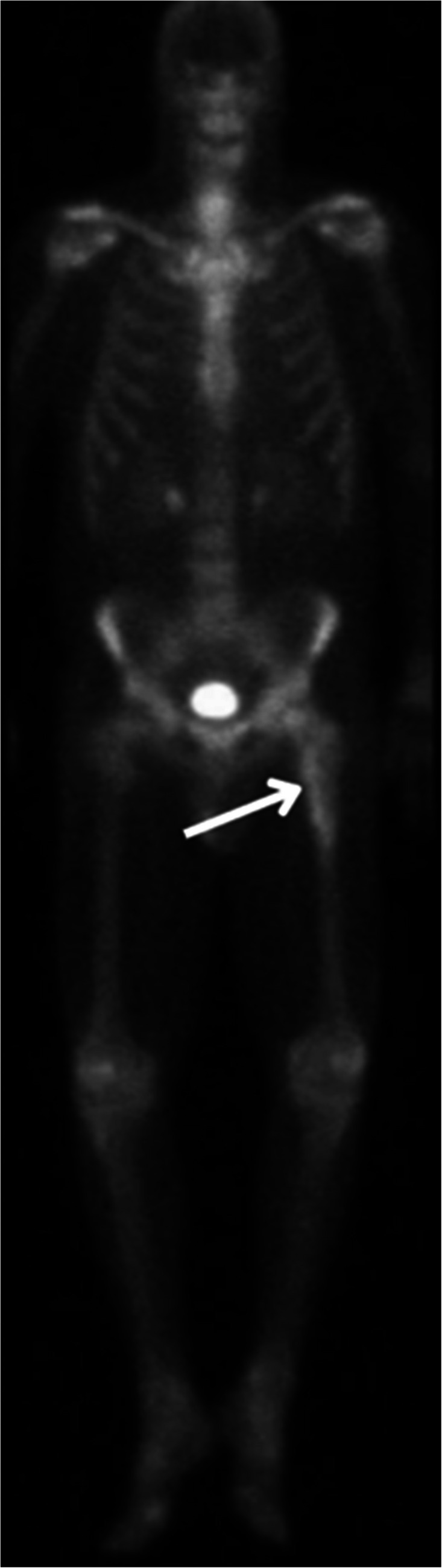
Anterior-posterior whole body view from bone scintigraphy reveals an elongated area of increased uptake of radionuclide extending from subchondral proximal femur to the proximal diaphysis with a flame shape inferiorly

Initial radiographs demonstrated a confluent radiolucent lesion extending from the proximal femur involving the intertrochanteric and subtrochanteric region to the diaphysis with a sharply demarcated, flame-shaped distal border and endosteal resorption, suggestive of the osteolytic phase of Paget’s disease (PBD) in long bones (Fig. 1A). Additionally, mild cortical thickening and trabecular coarsening were noted, further suggesting this diagnosis. However, the absence of significant bone expansion and the presence of permeative destruction on computed tomography (CT) raised concern for an alternative pathology. Magnetic resonance imaging (MRI) findings showed diffuse marrow replacement, cortical erosion, periosteal elevation, and extension into the adjacent proximal thigh soft tissues (Fig. 2). Whole body bone scintigraphy showed increased uptake of radiotracer in the left proximal femur extending from subchondral bone to the proximal femur with a flame-shaped area distally, corresponding to the lytic area seen on CT and radiographs (Figs. 3 and 4). No additional focal areas of increased uptake were evident.

Accentuation of a coarse trabecular pattern with flame-shaped areas on radiographs was suggestive of the lytic phase of Paget’s disease, but the MRI findings of cortical destruction and soft tissue extension were considered outside of the normal spectrum of PDB. Because of these atypical features for PDB of bone, there were concerns for other diagnostic considerations including primary lymphoma of bone. As a result, the patient underwent an open biopsy under general anesthesia by orthopedic surgery. Histology from this biopsy indicated a small round blue cell tumor consistent with lymphoma. Given concern for an impending pathological fracture due to extensive cortical involvement, and in the absence of a primary bone sarcoma, the patient underwent placement of a prophylactic intramedullary nail of the left femur after the frozen tissue evaluation [2].

Tissue cores were obtained during the biopsy procedure and sent to pathology for flow cytometry and diagnostic confirmation. Histopathology on permanent sections showed diffuse infiltrate composed predominantly of small lymphocytes. Immunohistochemical staining showed CD20, CD5, and CD23 positivity, with Cyclin D1 negativity, confirming a diagnosis of small lymphocytic lymphoma (SLL) as a result of chronic lymphocytic leukemia (CLL) [5]. Fluorescence in situ hybridization (FISH) analysis revealed deletion 13q and monosomy 17, along with deletion 17p, which is associated with an unfavorable prognosis due to TP53 disruption.

Subsequently, the patient was referred to radiation oncology and hematology-oncology. He was started on BTK (Bruton’s tyrosine kinase) inhibitor and acalabrutinib maleate 100 mg PO BID and underwent concurrent external beam radiation therapy to the left femur at a dose of 2400 cGy in 12 daily 200 cGy fractions. Radiation treatments were tolerated well and completed.

**Fig. 5:**
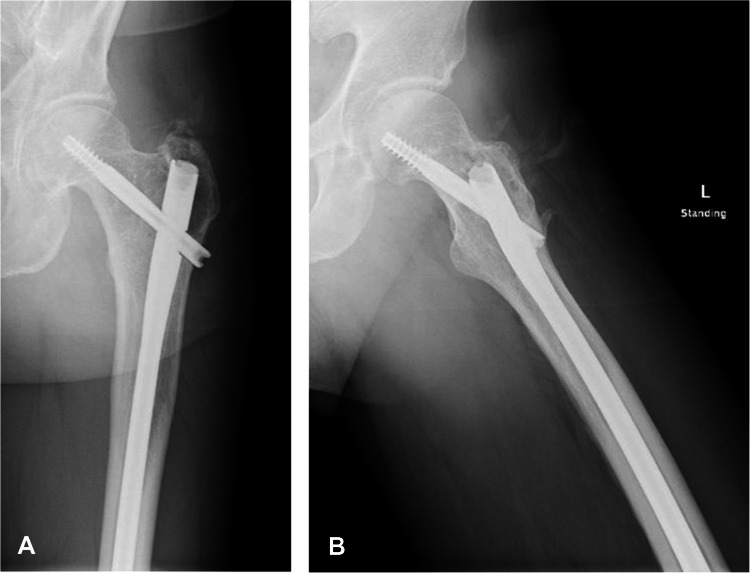
**A**, **B** Anteroposterior and lateral radiographs of the proximal and distal femur show significant improvement in the proximal diaphyseal femoral lesion, with reduced radiolucency and evidence of healing

The patient returned for a 9-month follow-up after biopsy and prophylactic stabilization. He reports feeling back to normal and has resumed regular activities and work. Radiographs show significant improvement in the radiolucency of the diaphyseal femoral lesion, with no evidence of recurrent or new pathology. There is progressive cortical thickening around the entire intramedullary defect, indicating a healing response. Previously noted substantial endosteal scalloping in this region has also improved (Fig. 5). The patient remains on acalabrutinib, managed by the medical oncology department, with no reported adverse effects.

## Discussion

Paget’s bone disease and bone lymphoma (in this case, SLL) can present with similar clinical and radiographic manifestations, including pain, radiolucent bone lesions, and pathologic fractures [2]. In this case, radiographs and CT highly suggestive of PBD were called into question based on the MRI results. Subsequent biopsy confirmed the diagnosis of lymphoma, which emphasizes the need to consider lymphoma in the differential diagnosis of PBD.

CLL and SLL are both types of non-Hodgkin’s lymphoma that affect B-lymphocytes. Both conditions are different manifestations of the same disease but differ in the localization of malignant lymphocytes. SLL refers to cancerous cells found predominantly in the lymph nodes. When these malignant cells circulate in peripheral blood or reside in the bone marrow, the condition is classified as CLL. Despite the regional differences, both are managed similarly and collectively referred to as CLL/SLL [6]. In the case with this patient, pathology was consistent with SLL.

On radiographs, PBD typically presents with an osteolytic appearance, coarse trabeculae, bone enlargement, trabecular and cortical thickening, often with a characteristic flame-shaped lesion (Fig. 1A). In contrast, osseous lymphoma such as SLL or CLL often manifests more aggressive patterns such as lytic lesions exhibiting permeative bone destruction, cortical thickening, and periosteal reaction. MRI findings in PBD can present abnormal marrow signal intensity, appearing as a low T1 and high T2 signal intensity. Soft tissue extension in a purported case of PBD, although exceedingly rarely reported in pseudosarcomatous change [7], should raise suspicion for a malignant process as opposed to PBD [8]. Likewise, bone scintigraphy of PBD and lymphoma may exhibit similar features of intense accumulation of radiotracer, making differentiation between the two a challenge.

As demonstrated in this case and previous studies, biopsy is not typically required for diagnosing PDB; it becomes essential only when imaging features raise concern for an alternate diagnosis such as an associated soft-tissue mass or suspicion for malignant transformation [7]. When lymphoma is suspected as a differential diagnosis, it is essential to obtain additional tissue cores at biopsy for specialized histologic analysis, including flow cytometry and immunohistochemistry. An open biopsy was elected as it allows for larger tissue samples to be obtained and is deemed to be more accurate in diagnosing malignancy [9].

In conclusion, mistaking lymphoma for Paget’s disease can lead to delayed oncologic intervention, potentially affecting patient outcomes. This case highlights the challenge in differentiating PDB and lymphoma. MRI was a valuable technique in this case where neither radiograph nor CT provided information that suggested the need for biopsy. Soft tissue extension in PDB is exceedingly rare, and its identification should raise suspicion for a more aggressive process and lower the threshold for obtaining tissue for definitive diagnosis.

## Data Availability

Imaging and other data used in this report are not publicly available due to privacy restrictions.
